# The changed functional status of the brain was involved in patients with poststroke aphasia: Coordinate‐based (activation likelihood estimation) meta‐analysis

**DOI:** 10.1002/brb3.1867

**Published:** 2020-10-06

**Authors:** Ying Du, Yujun Lee, Chuan He, Lihan Peng, Qian Yong, Zhiyi Cen, Yuqin Chen, Xin Liu, Xiaoming Wang

**Affiliations:** ^1^ Nursing Department Affiliated Hospital of North Sichuan Medical College Nanchong China; ^2^ Foreign Language Department North Sichuan Medical College Nanchong China; ^3^ Nursing Department North Sichuan Medical College Nanchong China; ^4^ Department of Cardiology Affiliated Hospital of North Sichuan Medical College Nanchong China; ^5^ Department of Neurology Affiliated Hospital of North Sichuan Medical College Nanchong China; ^6^ Department of Neurology Affiliated Hospital of Chengdu University Chengdu China

**Keywords:** ALE, aphasia, meta‐analysis, rs‐fMRI, stroke

## Abstract

**Background and Purpose:**

Although many functional magnetic resonance imaging (fMRI) studies have investigated the language architecture and neurobiological mechanism underlying poststroke aphasia (PSA), the pathophysiological mechanisms of PSA still remain poorly understood. In addition to a limited number of subjects (<20) tested with different methodologies and stimuli, inconsistent reports of the brain regions involved have been a major factor. Thus, we conducted a meta‐analysis of 12 peer‐reviewed studies of abnormal brain activation regions in PSA patients at rest using activation likelihood estimation (ALE).

**Results:**

A meta‐analysis was performed based on 24 experiments with 497 total participants in 12 studies to establish the ALE of regional activation in PSA. Through experiments with PSA patients and healthy controls, we found that hypoactivation in PSA converged on the left superior frontal gyrus and the left parietal postcentral gyrus, whereas there was hyperactivation in the right cerebellar anterior lobe, left fusiform gyrus, left superior parietal lobule, and right subgyral hippocampus.

**Conclusion:**

Our study verified that dominant and nondominant language networks play roles in the recovery of language function.

## Graphical abstract

Although the underlying pathophysiological mechanism of poststroke aphasia (PSA) is greatly concerned, it remains unclear. By coordinate‐based (ALE) meta‐analysis, we found that the activation of the left superior frontal gyrus (SFG) and the parietal postcentral gyrus of PSA presented hypoactivation, while there was hyperactivation in the right cerebellar anterior lobe, fusiform gyrus, superior temporal gyrus (STG), superior parietal lobule (SPL), and subgyral hippocampus. It is worth noting that the changed functional status of area in brain is more involved in right lobe. The future studies on rehabilitation should be given more attention to the right area.

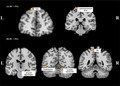

## 1 INTRODUCTION

Language impairment is one of the clinical manifestations of stroke. The estimated prevalence of poststroke aphasia (PSA) ranges from 21% to 38% (Berthier, 2005). Patients with PSA suffer not only isolated social emotions (Kauhanen et al., 2000), depression (Bullier et al., 2020), and low quality of life (Wang et al., 2018) but also speech impairment. Speech impairment may be improved by using cortical stimulation or pharmacotherapy. However, half of PSA patients recover much or all of their language function. The remainders are left with persistent and disabling impairment of communication (Geranmayeh et al., 2014).

With the advent of neuroimaging, many studies have investigated the abnormal regional activity and functional connectivity (FC) of PSA (Guo et al., 2019; Mayorova et al., 2019; Sandberg, 2017; Yang et al., 2016; Zhu et al., 2014). However, the results of these studies are inconsistent because of small samples (<20 patients) and variable analysis methods. For example, Yang et al. (2016) investigated the mechanism underlying PSA with a combination of structural alternations of gray matter volume and intrinsic functional MRI. They found that increased activation of remote interregional functional connection was located between the right IPL/SMG and right precuneus, right angular gyrus, and right superior occipital gyrus. The reduced activity was located at the right caudate gyrus and supplementary motor area and dorsolateral superior frontal gyrus. However, Fridriksson et al. (2010) studied brain activation associated with correct picture naming in 15 patients with aphasia. Their study revealed that the increased activation was preserved in the left hemisphere areas only. In their study, no reduced activity was found. These different findings are possibly due to the different research methods. Furthermore, Yang et al. (2016) examined changes in the local synchronization of spontaneous functional magnetic resonance imaging blood oxygen level‐dependent fluctuations in PSA at rest. They found that decreased intrinsic local synchronization was located in the right lingual gyrus, the left calcarine, the left cuneus, the left superior frontal gyrus (SFG), and the left medial of SFG in PSA patients. They also found that local synchronization in the left medial SFG was positively correlated with aphasia severity and naming scores on the Chinese Standard Aphasia Battery. In all, the results of neural imaging studies are hard to reach consensus.

To overcome this problem, we performed an activated likelihood estimation (ALE) meta‐analysis to study the convergence of the activated brain regions in PSA patients and HCs using rs‐fMRI. The ALE approach can be used to integrate the reported coordinates of activation maxima from published studies to identify regions of significant converging activation (Warren et al., 2009). This approach has two advantages: We can take whole pictures of brain activation differences between PSA patients and healthy controls, and we can study relatively larger samples. This study is expected to contribute to further understanding of the pathology of PSA.

## 2 MATERIALS AND METHODS

### 2.1 Literature search and selection criteria

In May 2020, the relevant studies were identified through a systematic online database search for peer‐reviewed articles on Web of Science, PubMed, and Elsevier. The searches were conducted with the keywords “stroke” or “apoplexy” or “cerebralvascular accident” or “cerebral infarction” or “cerebral haemorrhage”, “aphasia”, “resting‐state functional magnetic resonance imaging” or “rs‐fMRI”. The retrieval formula was (“stroke” [Mesh Terms] OR apoplexy OR cerebralvascular accident OR “cerebral infarction” [Mesh Terms] OR “cerebral haemorrhage” [Mesh Terms]) AND (“aphasia”[Mesh Terms]) AND (resting‐state functional magnetic resonance imaging OR rs‐fMRI).

**Figure 1:**
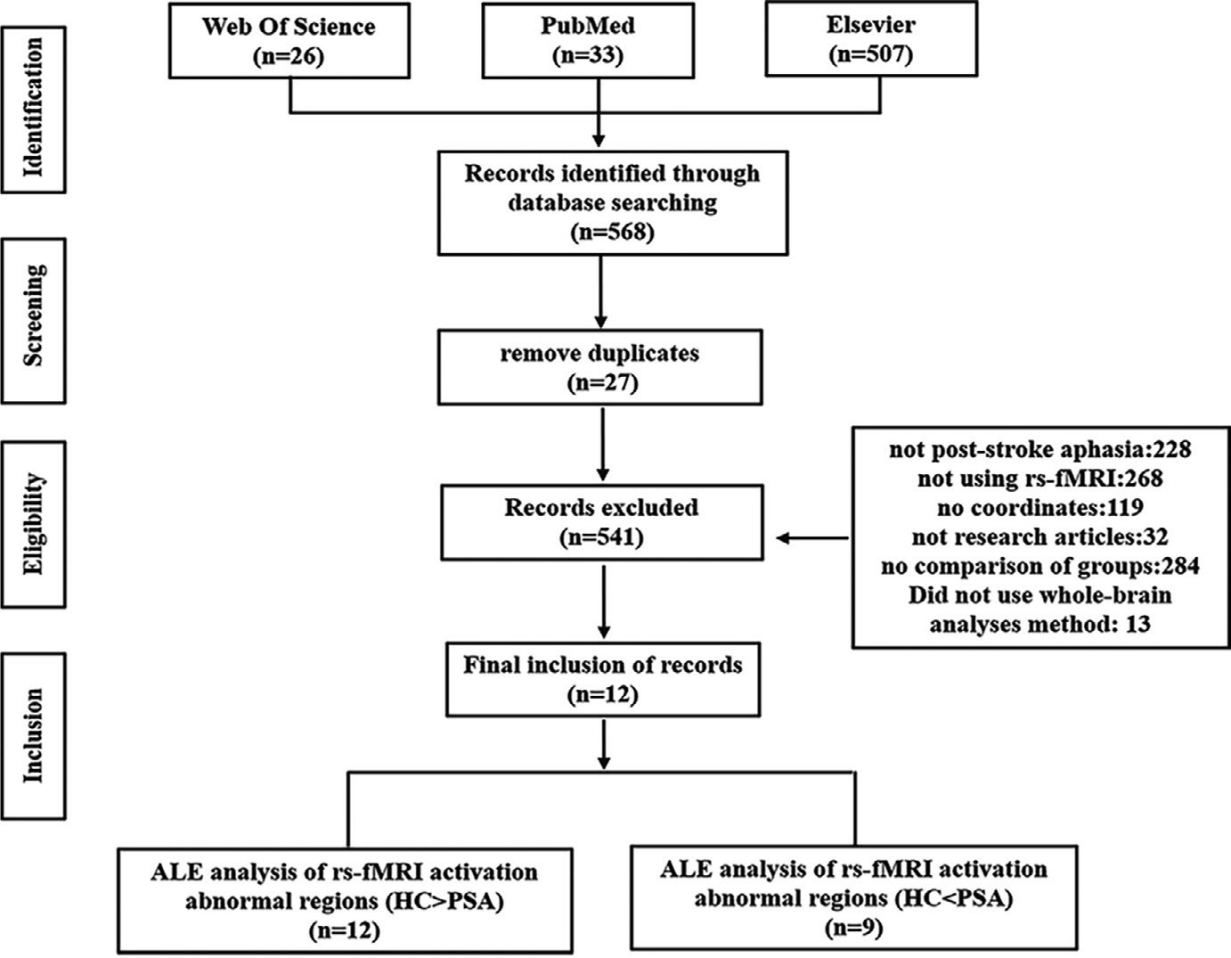
The literature search and the process of inclusion or exclusion of articles

First, 568 articles were obtained from the initial screening. Twenty‐seven duplicated articles were removed because they were duplicated. Then, 541 potential studies were further assessed according to the following criteria: (a) The study included PSA patients, (b) the study used rs‐fMRI, (c) the coordinates in the selected studies were in Montreal Neurological Institute (MNI) (Collins et al., 1994; Evans et al., 1993) space or Talairach space (Talairach, 1988), (d) the studies were written in English, (e) the studies were research articles, (f) the reports contain comparisons between the PSA group and health controls, and the PSA patients did not receive an intervention, and (g) quantitative whole‐brain analyses were performed. Reports with ROI analyses were excluded. Finally, a total of 12 relevant rs‐fMRI studies were included in the final meta‐analysis, which included 270 healthy participants and 227 stroke survivors. Table 1 illustrates the socio‐demographic and clinical characteristics of the subjects included in the selected studies. The retrieval process of each selected study is shown in Figure 1, and the methodological characteristics are shown in Table 2.

**Table 1:** Socio‐demographic and clinical characteristics of the subjects included in the selected studies

Conditions/study	Subjects (*n*)	Gender (M/F)	Patients					
			Age (years)	Stroke type	Aphasia type	Poststroke time	Lesion size (mm^3^)	Treatment method
van Hees et al. (2014)	20	9/11	56.38 ± 9.15	Ischemic	—	52.25 ± 49.84d	67.715	Semantic/phonologically
Yang et al. (2016)	37	23/14	53.53 ± 14.06	Ischemic or Hemorrhagic	Broca's or conduction or anomic	9.9 ± 5.4d	95.19	—
Yang et al. (2016)	37	23/14	53.53 ± 14.06	Ischemic or Hemorrhagic	Anomic or Broca's or conduction	9.72 ± 5.30d	28.85 ± 42.84	—
Guo et al. (2019)	37	23/14	53.53 ± 14.06	Ischemic or Hemorrhagic	Broca's	9.72 ± 5.30d	30.60 ± 42.23	—
Wu et al. (2015)	30	18/12	63.4 ± 13.2	Ischemic	Broca's	15 days to 11.5 weeks	—	—
Chen et al. (2019)	97	50/47	57.86 ± 11.17	Ischemia or Hemorrhagic	Broca's	1 week to 3 weeks	6.10 ± 8.55	—
Yang et al. (2016)	37	23/14	53.53 ± 14.06	Ischemic or Hemorrhagic	Anomic and Broca's and conduction	9.72 ± 5.30d	28.85 ± 42.84	—
Zhu et al. (2014)	34	—	50–75	—	—	2 weeks to 1 month	—	—
Yang et al. (2016)	38	24/14	53.67 ± 13.66	Ischemic or Hemorrhagic	Broca's	9.72 ± 5.30d	30.60 ± 42.23	‐
Torres‐Prioris et al. (2019)	27	—	49 ± 5.29	Ischemic or Hemorrhagic	Fluent aphasia	13.67 ± 13.42d	—	SLT and physiotherapy
Guo et al. (2019)^]^	37	23/14	53.53 ± 14.06	Ischemic or Hemorrhagic	Broca's	9.72 ± 5.30d	30.60 ± 42.23	—
Zhao, Ralph, & Halai (2018)	66	—	63.40 ± 13.57	Ischemic or Hemorrhagic	Anomia or Broca's or mixed nonfluent or global or conduction	51.49 ± 50.12d	—	—

Abbreviations: *N*, number of subjects; SLT, speech language therapy.

**Table 2:** All studies entered into the meta‐analysis are listed, including year, first author, stereotactic space, neuroimaging, imaging method, and active contrast conditions

Conditions/study	Stereotactic space	Neuroimaging	Imaging method	Activate contrast conditions
HC > PSA				
van Hees et al. (2014)	MNI	rs‐fMRI	Whole‐brain analysis	Controls > aphasia pretreatment
Yang et al. (2016)	MNI	rs‐fMRI	Whole‐brain analysis	Control > patient in ReHo clusters
Yang et al. (2016)	MNI	rs‐fMRI	Whole‐brain analysis	HC > aphasia in low‐frequency fluctuation HC > aphasia in seed‐based functional connectivity
Guo et al. (2019)	MNI	rs‐fMRI	Whole‐brain analysis	HC > aphasia in abnormal short‐range FCD volume HC > aphasia in abnormal long‐range FCD volume
Wu et al. (2015)	MNI	rs‐fMRI	Whole‐brain analysis	Control > ischemic stroke patients
Chen et al. (2019)	MNI	rs‐fMRI	Whole‐brain analysis	HC > stroke patients in dynamic ALFF HC > stroke patients in dynamic ReHo
Yang et al. (2016)	MNI	rs‐fMRI	Whole‐brain analysis	HC > aphasia in abnormal gray matter volume HC > aphasia in intrinsic functional connectivity
Zhu et al. (2015)	MNI	rs‐fMRI	Whole‐brain analysis	HC > patients in ALFF
Yang et al. (2017)	MNI	rs‐fMRI	Whole‐brain analysis	HC > aphasia in FA
Torres‐Prioris et al. (2019)	MNI	rs‐fMRI	Whole‐brain analysis	HC > the three aphasic subjects in metabolic activity
Guo et al. (2019)	MNI	rs‐fMRI	Whole‐brain analysis	HC > aphasia in dALFF HC > aphasia in dFC
Zhao et al. (2018)	MNI	rs‐fMRI	Whole‐brain analysis	HC > aphasia in the patients' fluency abilities
HC < PSA				
van Hees et al. (2014)	MNI	rs‐fMRI	Whole‐brain analysis	Controls < participants in aphasia pretreatment
Yang et al. (2016)	MNI	rs‐fMRI	Whole‐brain analysis	HC < aphasia in low‐frequency fluctuation HC < aphasia in seed‐based functional connectivity
Guo et al. (2019)	MNI	rs‐fMRI	Whole‐brain analysis	HC < aphasia in abnormal short‐range FCD volume HC < aphasia in abnormal long‐range FCD volume
Wu et al. (2015)	MNI	rs‐fMRI	Whole‐brain analysis	Control < ischemic stroke patients
Chen et al. (2019)	MNI	rs‐fMRI	Whole‐brain analysis	HC < stroke patients in dynamic ALFF HC < stroke patients in dynamic ReHo
Yang et al. (2018)	MNI	rs‐fMRI	Whole‐brain analysis	HC < aphasia in abnormal gray matter volume HC < aphasia in intrinsic functional connectivity
Zhu et al. (2015)]	MNI	rs‐fMRI	Whole‐brain analysis	HC < patients in ReHo
Guo et al. (2019)	MNI	rs‐fMRI	Whole‐brain analysis	HC < aphasia in dFC
Zhao et al. (2018)	MNI	rs‐fMRI	Whole‐brain analysis	HC < aphasia in semantic abilities

*NOTE*:rs‐fMRI, resting‐state functional magnetic resonance imaging; MNI = Montreal Neurological Institute.

### 2.2 Activation likelihood estimation meta‐analysis

An ALE meta‐analysis was performed using GingerALE software (Eickhoffet al., 2012; Turkeltaub et al., 2012) using the most recent users’ manual (http://brainmap.org/ale/manual.pdf). In the ALE meta‐analysis of single datasets, we connected the coordinates from the rs‐fMRI studies showing “HC > PSA” and “HC < PSA.” The ALE map was computed with *p* < .05 FEW cluster‐corrected thresholds with 1,000 permutations (Collins et al., 1994). According to the manual, this threshold is the most conservative and appropriate level. Resulting ALE maps were visualized by Mango software 4.0.1 (The University of Texas, Austin TX, USA) with the Colin brain template in MNI space (http://brainmap.org/ale/).

## 3 RESULTS

### 3.1 Brain regions with greater activation among HCs than among PSA patients

**Figure 2:**
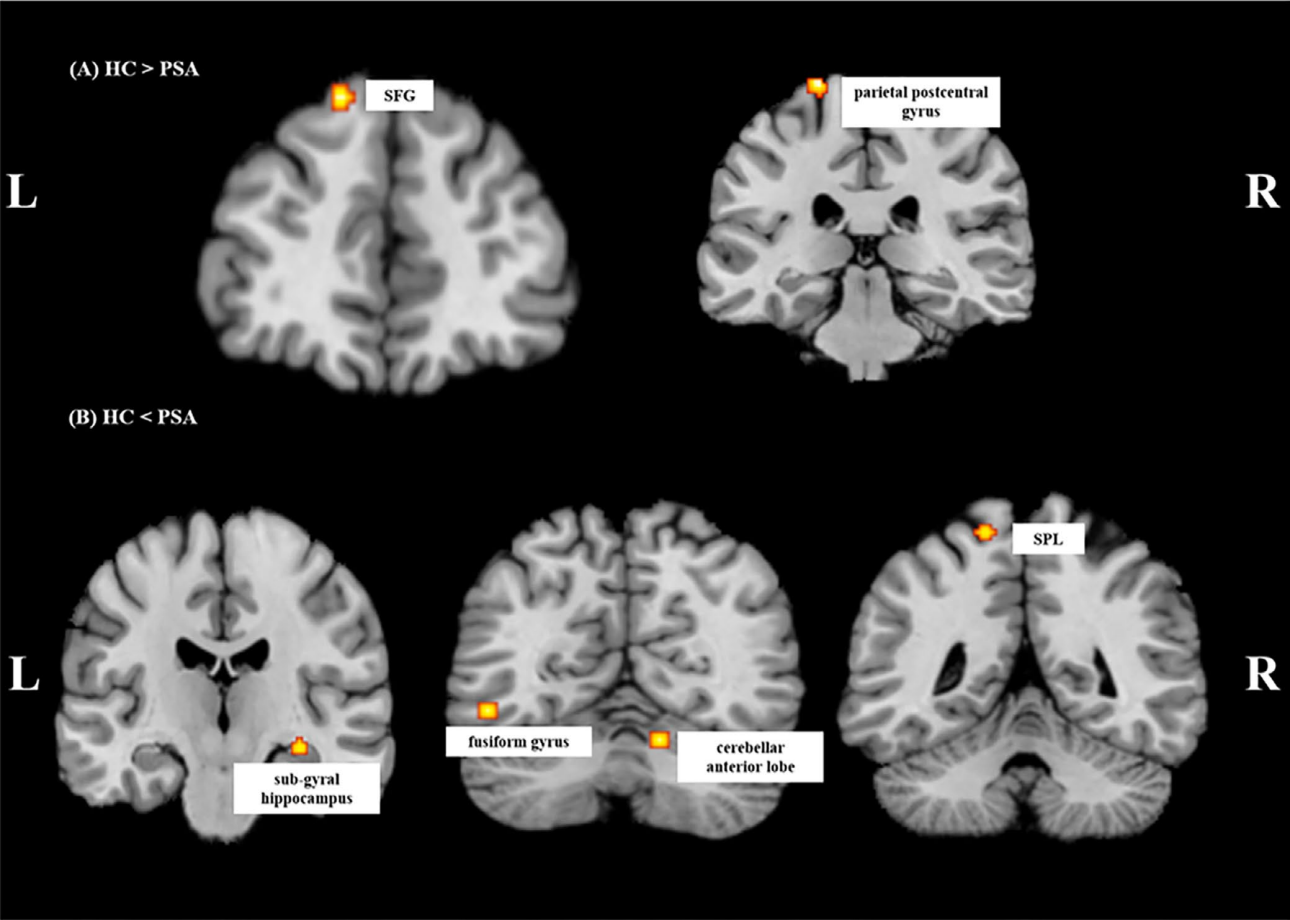
Significant results from the meta‐analysis of HC > PSA and HC < PSA during rs‐fMRI brain activation (FWE‐corrected < 0.05). SFG, superior frontal gyrus; SPL, superior parietal lobule. Peak coordinates (*x*; *y*; *z*) in the MNI stereotactic space are presented. L, left; R, right

Compared with the HCs, PSA involved lower activation in the left SFG (BA8, *x* = −12, *y* = 44, *z *= 48) and the left parietal postcentral gyrus (BA3, *x* = −20, *y* = −30, *z *= 78) (see Table 3 and Figure 2).

**Table 3:** Results of ALE analysis of different brain activation regions with HC > PSA and HC < PSA during resting‐state functional studies

Cluster #	Volume (mm^3^)	Brain region	BA	Side	MNI coordinates			*Z* score
					*X*	*Y*	*Z*	
HC > PSA	96	SFG	8	L	−12	44	48	5.64
	336	Parietal postcentral gyrus	3	L	−20	−30	78	7.57
HC < PSA	16	Cerebellar anterior lobe	—	R	12	−63	−24	4.94
	16	Fusiform gyrus	37	L	−48	−64	−12	4.94
	408	SPL	7	L	−16	−48	66	7.15
	128	Subgyral hippocampus	—	R	32	−20	−12	5.65

*Abbreviation:* ALE maps were computed at a familywise error (FWE)‐corrected threshold of *p* < .05, with a minimum cluster size of *k* > 10 mm^3^.

### 3.2 Brain regions with lower activation among HCs than among PSA patients

As demonstrated in Table 3 and Figure 2, the direct comparison between HCs and PSA patients revealed that the latter had more activation peaks in the right cerebellar anterior lobe (*x* = 12, *y* = −63, *z *= −24), left fusiform gyrus (BA37, *x* = −48, *y* = −64, *z *= −12), left SPL (BA7, *x* = −16, *y* = −48, *z *= 66), and right subgyral hippocampus (*x* = 32, *y* = −20, *z *= −12).

## 4 DISCUSSION

In the present study, ALE meta‐analysis was used to compare brain activation regions involved in PSA relative to HCs in rs‐fMRI. The first analysis of coordinates of resting‐state activation in PSA patients showed converged hypoactivation in the left SFG and left parietal postcentral gyrus. The second analysis found converged hyperactivation of brain areas including the right cerebella anterior lobe, left fusiform gyrus, left SPL, and right subgyral hippocampus.

The decreased activation in the left SFG (BA8 and left parietal postcentral gyrus (BA3) has been reported in several functional neuroimaging studies on stroke patients (Kang & Kim, 2008; Tohgi et al., 1995). The SFG was used for spatial analysis of working memory (Boisgueheneuc et al., 2006) and was used to process phonological or vocabulary search tasks (Tohgi et al., 1995; Tonkonogy & Goodglass, 1981). In our results, the reduced activation of the SFG in PSA was consistent with the patients' speech fluency and lexical dysphonia. The parietal postcentral gyrus has been considered an important region of somatosensory (Corkin et al., 1970) and writing abilities (Magrassi et al., 2010). Furthermore, in terms of FC, Gómez, Flores, and Ledesma (2007) found a strong correlation between the left parietal postcentral gyrus and the SFG, which suggests that frontoparietal networks in extensive processing of information during language production.

Converged hyperactivation was observed in the left fusiform gyrus (BA37) and subgyral hippocampus. A similar result has been found in previous rs‐fMRI studies that observed the activated regions during the changes to 17 PSA brain functions (Yang et al., 2016). Based on visual picture‐naming and auditory‐naming tasks to evaluate the cortical structure and dynamics of lexical semantic networks in driving speech production, the authors found that a distinct neuroanatomical substrate in the fusiform gyrus provided access to object semantic information (Forseth et al., 2018)]. Furthermore, the authors found that compared with HCs, PSA patients displayed hyperactivation of the left fusiform gyrus in a resting state, which may be related to the compensatory mechanism of semantic processing. The correlation suggested that the activity in the contralateral parahippocampus may mediate functional recovery of the right homologous language regions (Liégeois et al., 2004). Thus, the fusiform gyrus is very important for aphasia language recovery.

Converged hyperactivation during a resting state in SPA was also observed in the cerebellar right anterior lobe and the SPL. A previous rs‐fMRI study, which found increased activation of the bilateral cerebellum and ipsilesional superior parietal lobe during measuring the dynamic amplitude of low‐frequency fluctuations (dALFF) in patients with subacute stroke, reported similar results (Chen et al., 2019). The cerebellum was involved in the articulation, visual space, and executive power (Stoodley & Schmahmann, 2010), whereas the parietal lobe was associated with writing and cognitive function (Bzdok et al., 2016; Magrassi et al., 2010). A study that measured brain metabolism in PSA showed diffuse hypometabolism in the ipsilateral parietal area and contralateral cerebellum (Kim et al., 2012). Such results suggested that the cerebellum and parietal activation indicate the patients’ potential for further language improvement. Therefore, the right cerebellar anterior lobe and the SPL were closely related to language recovery in PSA.

## 5 LIMITATIONS

This study had several limitations. First, this study is a meta‐analysis of limited literature and may be affected by potential publication bias of invalid results. Second, ALE analysis cannot clarify the temporal dynamics of language changes in PSA patients. Third, this study included the literature using rs‐fMRI and did not include PET research. Therefore, future studies should strive to incorporate PET‐reported brain activation coordinates to explore abnormal activation regions in real‐world situations closer to aphasia patients.

## 6 CONCLUSION

The present study employed a meta‐analysis approach to map the convergence of abnormally activated brain areas in PSA. Our ALE meta‐analysis revealed that compared with HCs, PSA hypoactivation converged in the SFG and parietal postcentral gyrus, whereas hyperactivation occurred in the right cerebellar anterior lobe, fusiform gyrus, SPL, and subgyral hippocampus. Our study verified that dominant and nondominant language networks play roles in the recovery of language function. This study contributes to a better understanding of brain activation in PSA in the resting state. Moreover, our findings play an important role in understanding the pathophysiological mechanism of aphasia, creating rehabilitation strategies and predicting the effect of rehabilitation.

## DISCLOSURES

There was no conflict of interest to declare.

## AUTHOR CONTRIBUTION

Ying Du took part in the whole process of research and was responsible for literature search and essay writing. Yujun LEE was responsible for research design, data process, and data analysis. Chuan He Qian Yong and Zhiyi Cen were responsible for collecting data. Lihan Peng was responsible for data checking. Xin Liu was responsible for choosing literature. Yuqing Chen and Xiaoming Wang were responsible for guiding research design.

### Peer Review

The peer review history for this article is available at https://publons.com/publon/10.1002/brb3.1867.

## Data Availability

The data that support the findings of this study are openly available in Mendeley Data at http://dx.doi.org/10.17632/mmstgynxcp.2
